# Risk factors for reoperation due to periprosthetic joint infection after elective total hip arthroplasty: a study of 35,056 patients using linked data of the Swedish Hip Arthroplasty Registry (SHAR) and Swedish Perioperative Registry (SPOR)

**DOI:** 10.1186/s12891-022-05209-9

**Published:** 2022-03-23

**Authors:** Maria Qvistgaard, Jonatan Nåtman, Jenny Lovebo, Sofia Almerud-Österberg, Ola Rolfson

**Affiliations:** 1 Linnaeus University, Faculty of health and Life Sciences, Universitetsplatsen 1, 352 32 Vaxjo, Sweden; 2 Swedish Joint Arthroplasty Register, Gothenburg, Sweden; 3 Department of Research and development, Kronoberg County council, Vaxjo, Sweden; 4 Department of Orthopedic, Institute of Clinical Sciences, The Sahlgrenska Academy, University of Gothenburg, Gothenburg, Sweden

**Keywords:** Periprosthetic joint infection, Operative time, Prevention, Reoperation, Total hip arthroplasty

## Abstract

**Background:**

In Sweden, the incidence of a prosthetic joint infection (PJI) after a planned Total Hip Arthroplasty (THA) is 1.3%, but the worldwide incidence of PJI after THA is unknown. This study explores associations between reoperation due to PJI and potential risk factors.

**Methods:**

Primary elective THA surgery registered in both the Swedish Hip Arthroplasty Registry (SHAR) and the Swedish Perioperative Registry (SPOR) between 1 January 2015 and 31 December 2019 were included in this registry study, resulting in a total study population of 35,056 cases. The outcome variable was reoperation as the result of PJI within a year after surgery. Data were analysed using a multivariable Cox regression model.

**Results:**

Reoperation due to PJI occurred in 460 cases (i.e., 1.3% of the study population). Each year of age increased the risk with 2% (HR 1.02 Cl 1.01, 1.03 *P* = < 0.001). Compared to men, women had significantly less risk for reoperation (HR 2.17 Cl 1.79, 2.53 *P* = < 0.001). For patients with obesity (BMI > 30), the risk increased considerably compared to underweight, normal weight, or overweight patients (HR 1.89 Cl 1.43, 2.51 *P* = < 0.001). The risk also increased by 6% for every 10 min of operative time (HR 1.06 Cl 1.02, 1.09 *P* = < 0.001). Patients having general anaesthesia had greater risk compared to those with spinal anaesthesia (HR 1.34 Cl 1.04, 1.73 *P* = 0.024). Finally, a lateral approach showed higher risk of reoperation than a posterior approach (HR 1.43 Cl 1.18, 1.73 *P* = < 0.001).

**Conclusion:**

Recognition of the several risk factors identified in this study will be important for the perioperative management of patients undergoing THA.

## Background

Total Hip Arthroplasty (THA) is a cost-effective surgical intervention for severe hip joint pain caused mainly by osteoarthritis [1]. In Sweden, the risk of a prosthetic joint infection (PJI) after a planned THA is 1.3% [2]. The worldwide incidence of PJI after THA is unknown and probably underreported [3]. For example, some cases of aseptic loosening may be caused by infection [4] although not registered as such. One-year mortality rate after reoperation caused by PJI is 4.2%, and 5-year mortality is as high as 21.2% [5].

Risk factors for PJI include male, comorbidity, smoking, prolonged operative time, and a Body Mass Index > 30 (BMI, kg/m^2^). Patients with obesity class 1 (BMI 30–34.9) have twice as a high risk for PJI, and BMI > 40 increases the risk of PJI as much as four times [6]. Furthermore, being male increases the risk for PJI [7], but the specific reasons for this increase are not clear.

Smoking is also associated with higher rates of Surgical Site Infection (SSI) [8]. One study [9] found that adverse outcomes were reduced most in a group of smokers who had recently quit smoking, evidence that suggests smoking cessation should be a requirement or strong recommendation before surgery. Although smoke cessation compliance is difficult to assess, a surgeon’s kind but sharp request to cease smoking could motivate patients to stop smoking.

Identification of high-risk patients and risk factors within the surgical environment is a first step in preventing PJI. Despite assiduous preventive measures and technical developments (e.g., clean air suites, effective ventilation systems, and improved surgical techniques), the risk of PJI after THA has been constant for many years [10]. The constant level of PJI in spite of improved surgical conditions may partly depend on expanded patient group relevant for surgery [11]. PJI is an economic burden for health care and society in general as it requires extended care and use of hospital resources [12]. In addition, PJI often requires long-term treatment with antibiotics, which constitutes a risk for development of resistance [13]. PJI can cause a great deal of suffering, possibly negatively affecting a patient’s whole life situation [14].

This study explores associations between reoperation due to PJI and potential technical, individual, and environmental risk factors, such as operative time, type of anaesthesia and surgical approach, among several others.

## Methods

### Study design

This observational study uses prospectively collected data from two Swedish quality registries: Swedish Hip Arthroplasty Registry (SHAR) and Swedish Perioperative Registry (SPOR).

### Sources of data

Started in 1979, the SHAR is a quality registry that includes the specific details on implants as well as more general data such as age, sex, and BMI. During the study period, the completeness of SHAR was 98% [2]. Completeness is defined as the proportion of the procedures reported to the registry [15].

Started as a project in 2011 with the first annual report in 2015, SPOR contains a considerable amount of specific data concerning each surgical procedure not included in SHAR (e.g., choice of anaesthesia, type of ventilation, and exact time for start and end of each surgical procedure). The completeness of SPOR increased considerably during the study period, from 30% in 2015 to 98% in 2019 [16].

In SHAR, each surgical procedure is registered to an Operating Room (OR), but data for ventilation type were insufficient. Therefore, type of ventilation in each OR was determined by contacting each hospital. Two types of ventilation were found: turbulent mixing airflow, which is based on the principle of dilution, and unidirectional airflow, which uses parallel streamline flows to decrease turbulence and to prevent mixing with the surrounding air.

### Patient selection

Inclusion criteria were all patients over 18 years old who had gone through primary THA surgery from 1 January 2015 through 31 December 2019 in Sweden and were registered in both the SHAR and the SPOR. Individuals registered in both registries were linked using their ten-digit personal identity number. Patients were excluded if the diagnostic indication for surgery was fracture (pelvis/hip), sequelae after fracture (pelvis/hip), or tumour. Cases with unlikely operative time (< 20 min and > 240 min) were also excluded. A few cases were excluded due to missing data concerning the OR ventilation type. For patients who had surgery (THA) on both hips during the study period, the first performed procedure was excluded [17].

### Data analysis and statistics

All patients were followed from their primary operation until the first reoperation due to PJI or reoperation due to other causes, death, or end of follow-up (1 year after the primary operation).

Descriptive statistics are presented as means with standard deviations or counts with percentages. The Kaplan-Meier estimator was used to study the rate of PJI over time. The association between possible risk factors and reoperation due to PJI was analysed using the multivariable Cox regression model. From this model, we derived *P* values and hazard ratios with 95% confidence intervals; *P* 0.05 was considered statistically significant.

For the patients who had missing data for at least one variable (20.1%), missing values were replaced using multivariate imputation by chained equations [18]. The imputation model included all predictor variables along with the Nelson-Aalen estimator of the baseline cumulative hazard and the outcome indicator. Five imputed datasets were generated and the analyses were based on the pooled estimates. A complete case analysis and sensitivity check were also performed. The results show no major differences of estimates. Data were analysed using R version 4.0.2 (R Foundation for Statistical Computing, Vienna).

## Results

**Fig. 1:**
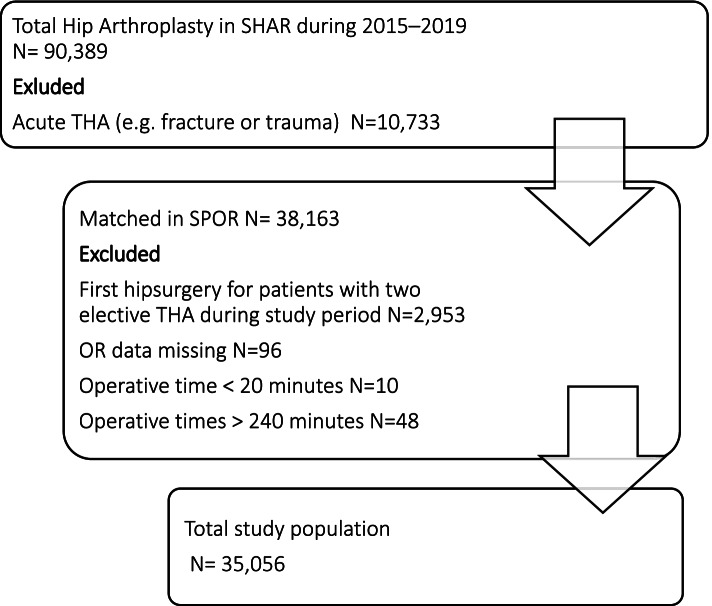
Flowchart. Flowchart of included patients in this study

Of the 79,656 cases eligible for inclusion in SHAR, 38163 cases were registered in SPOR. Figure 1 depicts a flow chart of the process for excluding cases. The final study population was 35,056 cases.

Mean age for all patients was 69 years, and most patients (50.7%) were between 70 and 84 years old. The majority of patients were women (57.6%). The population had the following American Society of Anaesthesiologists Physical Status Classification (ASA-class): ASA I (17.4%); ASA II (60.4%); and ASA III and IV (22.2%). Mean BMI (kg/m^2^) was 27.5, 30.5% of the patients were normal or underweight, 21.3% of the patients were in the obesity classes 1 (BMI 30–34.9), 6.4% were in obesity class two or three (> 35). A Major part of the patients had their surgery at a county hospital (81.4%). Most patients (86.7%) received spinal anaesthesia during surgery. Posterior approach was slightly more common (53.5%) than lateral approach (45.4%). Fixation with bone cement of both components was 59.6%, hybrid fixation was 15.7%, and uncemented fixation was 24.7%. The vast majority of the surgical procedures (84.9%) were performed in operating rooms with unidirectional airflow. The mean operative time for THA was 85 min (cemented: 90 min; uncemented: 77 min; and hybrid: 81 min). Reoperation as the result of PJI occurred in 460 cases (1.3%). Descriptive statistics are presented in Table 1.

**Table 1:** Demographics

Factor	Categorization	Numbers (%) if not stated otherwise	Missing (%)
***Patient characteristics***			
Sex	Male	14,869 (42.4%)	0.0
	Female	20,187 (57.6%)	
Age, mean (SD)		69.5 (10.5)	0.0
Age group	< 55	3228 (9.2%)	0.0
	55–69	12,303 (35.1%)	
	70–84	17,786 (50.7%)	
	85+	1739 (5.0%)	
ASA classification	ASA I	6087 (17.4%)	0.2
	ASA II	21,147 (60.4%)	
	ASA III and IV	7769 (22.2%)	
BMI, mean (SD)		27.5 (4.5)	1.1
BMI class			
*Normal weight and underweight*	< 18.5–24.9	10,555 (30.5%)	
*Overweight*	25–29.9	14,570 (42.0%)	
*Obesity class 1*	30–34.9	7303 (21.1%)	
*Obesity class 2/3*	> 35	2225 (6.4%)	
Smoking status	Non-smoker	28,797 (95.1%)	13.6
	Smoker	1484 (4.9%)	
Diagnosis	Primary osteoarthritis	32,097 (91.6%)	0.0
	Other	2977 (8.4%)	
Reoperation due to PJI within 1 year		460 (1.3%)	
***Intraoperative characteristics***			
Level of hospital	University hospital	2466 (7.0%)	0.0
	County hospital	28,553 (81.4%)	
	Private hospital	4037 (11.4%)	
Anesthesia	Spinal	28,361 (86.7%)	6.7
	General	4346 (13.3%)	
Incision	Lateral	15,905 (45.4%)	0.0
	Posterior	18,755 (53.5%)	
	Other	394 (1.1%)	
Fixation	Cemented	20,861 (59.6%)	0.2
	Uncemented	8631 (24.7%)	
	Hybrid	5502 (15.7%)	
Operative time, mean minutes (SD)			0.0
*Overall mean (SD)*		85.4 (28.9)	
*Operative time cemented mean (SD)*		90.2 (27.9)	
*Operative uncemented mean (SD)*		76.9 (28.7)	
*Operative time hybrid mean (SD)*		80.6 (29.1)	
Type of ventilation	Unidirectional airflow	29,762 (84.9%)	0.0
	Mixed turbulent airflow	5294 (15.1%)	

*SD* Standard Deviation, *ASA* American Society of Anesthesiologists Physical Status Classification System, *BMI* Body Mass Index, *PJI* Periprosthetic Joint Infection

Demographics for the studied population Numbers (%) unless otherwise specified (mean, SD)

**Fig. 2:**
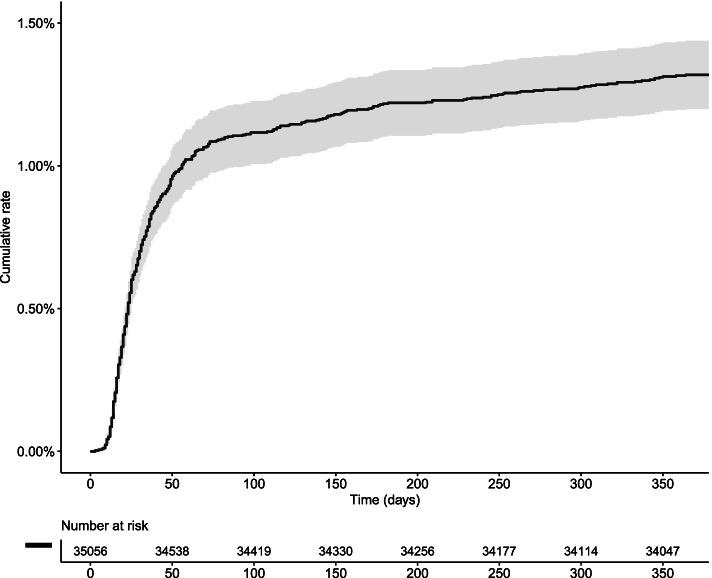
Kaplan-Meier curve. Cumulative incidence with help of 1- Kaplan-Meier estimate for reoperation caused by PJI

Most reoperations caused by PJI appeared within 75 days after surgery (Fig. 2). The scale below the figure shows the number at risk for PJI the first year after primary THA.

The results of the multivariate analysis show a statistically significant (*P* = ≤ 0.05) increase for risk of reoperation due to PJI in the following variables. Each year of older age increases the risk (HR 1.02 Cl 1.01, 1.03 *P* = < 0.001). Men have increased risk compared to women (HR 2.17 Cl 1.79, 2.63 *P* = < 0.001). Comorbidities have a negative impact of the risk or reoperation due to PJI (ASA-class II HR 1.56 Cl 1.10, 2.20 *P* = 0.012 ASA-class III/IV HR 1.59 Cl 1.08, 2.33 *P* = 0.018). Patients receiving general anaesthesia (HR 1.34 Cl 1.04, 1.73 *P* = 0.024) are at higher risk than patients receiving spinal anaesthesia. For patients with obesity, the risk increases considerably (HR 1.89 Cl 1.43, 2.51 *P* = < 0.001) compared to patients who are underweight, normal weight, or overweight. The lateral surgical approach increases the risk (HR 1.43 Cl 1.18, 1.73 *P* = < 0.001) compared to the posterior surgical approach. In addition, the risk for reoperation increases 6% every 10 min of operative time (HR 1.06 Cl 1.02, 1.09 *P* = < 0.001).

**Fig. 3:**
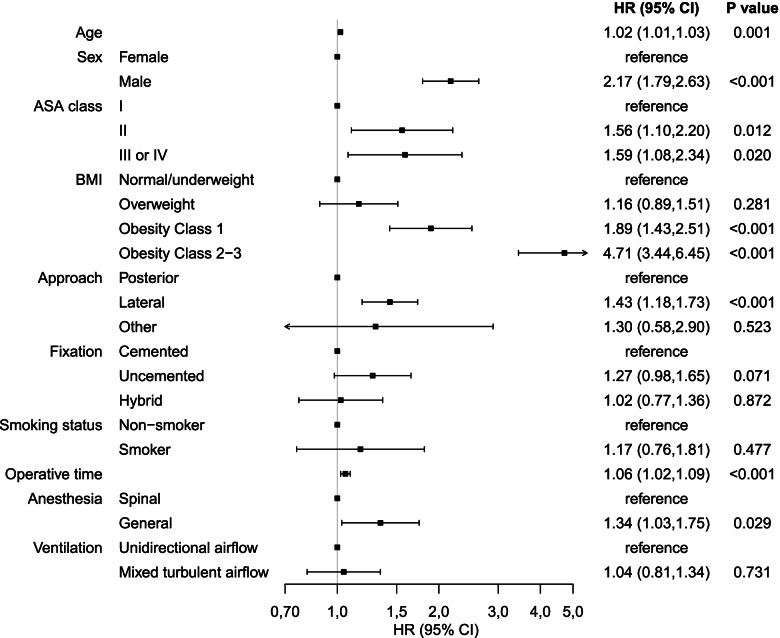
Forest plot. Results from multivariable Cox regression model

The following variables show no significance for increased risk of reoperation caused by PJI (*P* = > 0.05): components fixed without bone cement (HR 1.27 Cl 0.97, 1.67 *P* = 0.079) compared to both components fixed with bone cement; patients undergoing surgery in OR with mixed turbulent ventilation (HR 1.04 Cl 0.81, 1.34 *P* = 0.731) compared to surgery in OR with laminar ventilation; and smoker (HR 1.17 Cl 0.76, 1.81 *P* = 0.475) versus non-smoker. The full Cox regression model is presented as a forest plot in Fig. 3.

## Discussion

Significant results and other interesting aspects of the results will be discussed in this section. The results show correlation between the length of the operation for THA and the necessity for reoperation as the result of PJI. That is, long operative time constitutes a significant risk as each 10-min interval of surgery considerably increased the risk for reoperation. Other studies also confirm the risk of prolonged operative times related to complications [19, 20]. Overall, in various surgical procedures, the mean operative time was 30 min longer in operations followed by a SSI compared to those without SSI [20]. Time of exposure to air is a risk factor due to possible microbiological contamination of the wound [21] and therefore it should be short. “Chasing minutes” during a surgical procedure is multifaceted and must be carried out without comprising quality of care. For example, careful closure of the tissue layers reduces wound leakage [22]. However, operative time should never be longer than necessary. Experienced surgeons and surgical teams (i.e., high annual surgeon volume) is associated with less adverse events such as reoperation [23]. Typically, well-functioning surgical teams have clear strategies and skilled communication, characteristics associated with effective and safe care [24]. The cause of prolonged operative times need to be identified at each clinic performing THA.

In our studied population, the lateral surgical approach was associated with more reoperations as the result of PJI than the posterior surgical approach. A previous study [25] found that elective primary THA using the posterior surgical approach had a significantly lower rate of the most common complications (PJI, fracture, and prolonged wound drainage), although pain and functional outcomes were not considered. Both the lateral and posterior approach are commonly used worldwide when performing THA, and they both have their advantages and disadvantages. Choice of approach is closely related to the surgeon’s experience and comfort with one approach over another. The importance of the surgeon’s experience in a specific approach should not be underestimated [26].

The association between being overweight and PJI is difficult to determine. The linear association between BMI and PJI may not apply to THA. Both sex and age affect how being overweight can be used as a predictor for PJI. Surgeons need to consider each patient’s individual conditions to decide a reasonable limit for BMI [27]. The results in our study show that obesity classes 1–3 (BMI > 30) represent significant risk for reoperation as the result of PJI after THA. Therefore, patients should receive fact-based and transparent information about the risks associated with obesity. As individual consultation with a dietitian makes a small but significant difference in weight control [28], there could be value in studying the role of dietitians in weight management for this group of patients (i.e., patients with BMI > 30 who need THA). Moreover, weight loss needs to be controlled to avoid performing surgery on patients with poor nutrition status.

Clinical association between unidirectional airflow and decreased rates of PJI is still unclear and controversial. Overreliance on ventilation systems may weaken strict OR discipline, ultimately increasing rather than decreasing the risk [29]. However, a study based on data from the Norwegian Arthroplasty Registry showed lower risk for reoperation when surgery was performed in ORs with unidirectional airflow compared to conventional ventilation, which is the same as mixed turbulent airflow [30].

The increased risk for reoperation caused by PJI regarding age and ASA class were expected. ASA class defines morbidity well and with increased age more physical limitations come into play. It is clear that men are at higher risk than women for reoperation, but it is not clear why this is so. However, compared to women, men tend to have worse overall physical health, a fact that might explain why men have a higher risk for reoperation [31].

### Strengths and limitations

The main strength of registry studies in general is that data already exist and the research includes complete study populations [32]. The 35,056 cases in this study represent a total population even though the sample was reduced because the SPOR was incomplete during first and second year of data collection. Cases not registered in SPOR more often had their surgery at private clinics, were younger, were less morbid, and required fewer reoperations due to PJI. Variables with low coverage registered in SPOR were not used in this study.

This is the first study to link the data in SHAR and SPOR and therefore adds new valuable data to orthopaedic registry-based research.

All patients who met inclusion criteria and included in both registries between 2015 and 2019 were included. This study excluded patients with trauma and fracture due to THA, another strength of the study as it reduced heterogeneity. Higher rates of PJI within that group of patients could be a confounder due to different preoperative circumstances.

Ventilation type is reported annually to SHAR. This information was manually controlled to specifically control each operating room to a specific type of ventilation. Although this was time consuming, it ensured data were correct.

This registry study has some limitations. Change of practice and differences in registration may have varied over time, possibly affecting the outcome. In addition, as SPOR is relatively new, some variables had insufficient data. For example, we could not analyse number of people present inside OR during surgery, time interval between preoperative antibiotics and start of surgery, body temperature at the end of surgery, interesting aspects that need to be investigated when data are more complete. Follow-up of 1-year may be seen as a limitation. Longer follow-up may have strengthened data and the conclusions derived from its analysis.

## Conclusion

Prolonged operative time is a modifiable risk for PJI. The time a surgical wound is open should be as short as possible while maintaining the quality of the surgery. To counteract prolonged operative time, we propose striving for fixed surgical teams and keeping a high annual surgery volume. These practices should minimise unnecessary interruptions and extend operative time.

Depending on the results of high risk or reoperation caused by PJI, we also suggest that patients who need THA and have a BMI > 30 should be informed about the risks and offered individually-adjusted support regarding weight-control before surgery. Prevention is the most effective approach to avoid PJI in the long run and a sustainable way forward. Recognition of the risk factors identified in this study will be important for the perioperative management of patients undergoing THA.

## Data Availability

Datasets generated and analysed during current study are not publicly available due to controlled personal data agreement and data security but are available from the corresponding author on reasonable request.

## Abbreviations

*ASA-class*: American Society of Anaesthesiologists Physical Status Classification system
*BMI*: Body Mass index
*THA*: Total Hip Arthroplasty
*OR*: Operating Room
*PJI*: Periprosthetic Joint Infection
*SHAR*: Swedish Hip Arthroplasty Registry
*SPOR*: Swedish PeriOperative Registry
*SSI*: Surgical Site Infection
